# Ethno-medicinal survey of important plants practiced by indigenous community at Ladha subdivision, South Waziristan agency, Pakistan

**DOI:** 10.1186/s13002-016-0126-7

**Published:** 2016-11-15

**Authors:** Muhammad Abdul Aziz, Muhammad Adnan, Amir Hasan Khan, Atiq Ur Rehman, Rahmatullah Jan, Jafar Khan

**Affiliations:** 1 Department of Botany, Kohat University of Science and Technology, Kohat, 26000 Pakistan; 2 Department of Botany, Shaheed Benazir Bhuto University Sheringal, District Dir (Upper), Khyber Pakhtunkhwa, Pakistan; 3 Department of Botany, University of Science and Technology, Bannu, Pakistan; 4 Department of Botany, Islamia College , Peshawar, Pakistan

**Keywords:** Medicinal plants, Traditional knowledge, Ailments, Herbal therapies, Use value

## Abstract

**Background:**

Medicinal flora plays a vital role in treating various types of ailments in living beings. The present study was planned to investigate and document systematically the indigenous knowledge in a scientifically little explored area of Ladha sub-division, South Waziristan agency, Pakistan. Hence, this study would contribute positively to the field of ethnopharmacology.

**Methods:**

Prior to ethnomedicinal data collection, regular field visits were conducted during the month of May and June 2015 to locate the sites and respondents from where the traditional knowledge was to be recorded. Ethno-medicinal data was collected during the month July and August 2015 through rapid appraisal approach (RAA) based on direct interaction with the indigenous communities by making group discussions, corner meetings and semi-structured interviews. Data was evaluated statistically by using the index of Use value (UV) and Frequency of citations (FC).

**Results:**

A total of 82 medicinal plants belonging to 42 families were reported in the study. Leaves were the most frequently used plant parts. Highest use values were recorded for *Peganum harmala* (0.93), *Punica granatum* (0.91), *Thymus mongolicus* (0.90), *Chenopodium album* (0.89), *Coriandrum sativum* (0.87), *Mentha longifolia* (0.87), *Lactuca serriola* (0.87) and *Portulaca oleracea* (0.87). Medicinal plants used for the gastro intestinal complexities and respiratory diseases were more than 9% followed by skin and diarrhea (7% each), liver disorders (5%) cough and cold fever (5%).

**Conclusion:**

People of the area mostly still rely on traditional herbal therapies. Keeping in mind the dependence of the indigenous community for their primary health care on such herbal remedies, pharmacological and critical toxicological investigation of certain flora is necessary. Moreover, projects should be designed to analyze the existing issues and problems related with medicinal plants conservation.

## Background

Documentation of ethno-medicinal information have substantial role in illuminating folk knowledge, which facilitates the discovery of modern allopathic drugs [1, 2]. In modern pharmacopoeia, several synthetic drugs of plants’ origin have been documented. Currently in the developing countries, about 80% of the world’s populations rely on these traditional therapies to cope with several ailments [3–5]. Medicinal plants comprising of several biologically active factors [6]. Varieties of therapeutically active plants are used in herbal medicines, and have proved their efficacy to compete with the modern allopathic drugs [7]. Out of the total 265,000 flowering plants species [8], only a small proportion (5000 species) has been analyzed for their biological potential [9].

In Pakistan, traditional uses of of medicinal plants has been documented from many areas [10–16] but still in remote areas (including tribal areas) there is scarcity of reports, in which the folk knowledge about the medicinal plants have been properly mentioned [17–21]. Local people of certain areas utilize plants for their health maintenance because of poorer economic values and lack of modern health facilities [22]. These traditional medicines have been used for long time but unfortunately these valuable knowledge has been not properly recognized and documented in many areas of the country especially in federally administrated tribal areas (FATA) including South Waziristan agency.

Ladha is the sub-division of South Waziristan Agency. The introduction of allopathic medicines has greatly affected the knowledge, faith and skill about the traditional herbal therapies in the study area. Apart from these, the territory is also under critical condition due to armed conflict and other terroristic activities for the last one decade. Consequently middle class sect prefers to migrate partially or wholly to settle areas where the chances of exposure to modernization became more prominent which ultimately has negative effect on the consistency of traditional knowledge, losing its originality and going to the periphery of extinction. That is why the present study was planned to investigate, catalogue and record the folk knowledge and ethno-medicinal values of the local flora at Ladha so as to preserve the Ethno-medicinal knowledge and share it with other communities.

## Methods

### Study area

The undertaken study was carried out in Ladha, being a part of Federally Administered Tribal Areas (FATA). The study area comprised of a mass of rugged and complex hills and ridges. The overall area of the agency is comprised of 6619 km^2^ and is laying at 321 24′ 50″ N latitude and 691 42′ 06″ E longitude having 4100–7000 ft altitude. Temperature in the area falls to 0 °C during winter at some places with higher altitudes where snow fall also occurs. The winter is extremely severe with coldest months of December to February. The average rainfall per annum is 6 in. while in plain areas the summer season is comparatively much hot. Ethnographically two tribes are the inhabitants of the area ie Mehsud and Barki. The Pashto language is used for the communication in the locality.

### Socioeconomic status of the indigenous communities

The overall socioeconomic status of the indigenous communities is comparatively poor. The sources of income of these communities are different ie government servants, farmers, drivers while some have their own business. Most of the people manage their income from domestic and foreign remittances and forest products. In the study area, frequently the indigenous communities have cattle herds in their homes. The cattle are also a source of livelihood for most of the people. Almost half of the respondents, selected and interviewed were illiterate (50.44%), whilst most of those with an education had merely up to primary (29.09%) which reflects the unavailability of standard educational institution in the area (Table 1). It was also observed that the literate sect in the study area was less conversant with respect to traditional knowledge and uses of medicinal plants as compared to illiterate one. Those places which have no proper communication with advanced areas, people still depend on medicinal flora to treat and combat with disease. If the cultivation and sustainable use of medicinal flora are promoted in the area, it will positively affect the socioeconomic status of the indigenous communities.

**Table 1:** shows gender, age group and literacy level frequency of the respondents

Category	Total	Percentage
Gender		
Man	85	75.22
Female	28	24.77
Age groups		
28–40	25	22.12
> 40	88	77.87
Educational attainment		
Illiterate	57	50.44
Primary	29	25.66
Middle	18	15.92
Secondary	9	7.96
Occupation		
House wives	28	100
Shopkeepers	14	12.38
Farmers	38	33.62
Labors	27	23.89
Primary teachers	15	13.27
Local healers	20	17.69

### Informant selection and ethno-medicinal data collection

Prior to ethno-medicinal data collection, regular field visits were made to locate the sites and to gather information about the respondents and their expertise in traditional knowledge. Being the local inhabitant, Mr. Muhammad Abdul Aziz was aware of those sites where there is a significant trend for the utilization of traditional herbal recipes in their daily routine for treating ailments. All the meetings and discussions were in local language ie Pashto. Meetings were conducted with the local representative (*Malik*) of the area to display the main theme and objective of the study. In the study area, *Malik* is considered to be the head of a tribe and is responsible for dealing the local matters. Without their recommendations, community involvement is not possible in such kind of field surveys and studies. The step was taken in order to acknowledge their co-operation and to develop the confidence about the provided knowledge so as to gain valuable information.

Ethno-medicinal data collection was carried out in midsummer ie from May 2015 to August 2015. During the month of May and June, the local respondents were targeted for interviews, while the Ethno-medicinal data was collected in the months of July and August. As the area is covered by snow fall and the winter season is very cold in the territory, mostly the medicinal flora is blooming in the month of June, July and August. All the selected informants were reported to be highly conversant about the traditional therapies but most of the data was taken from the local healers.

**Fig. 1:**
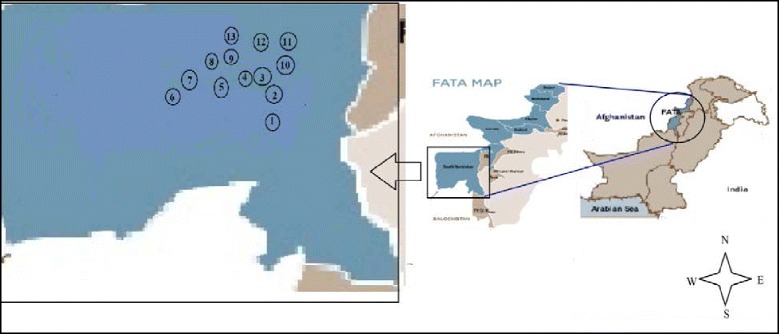
Map of the reported plants’ locations (number in circles). Locations are designated with numbers as 1-Landay Karama; 2-Karama; 3-Kacha Langer Khel; 4-Sam; 5-Kaniguram; 6-Chalwashtai; 7-Meeshta; 8-Mordar Algad; 9-Ashpashteen; 10-Malak Mella; 11-Speena Mella; 12-Zawar Klai; 13-Ladha

Rapid appraisal approach (RAA) was conducted to collect the indigenous knowledge. Survey was based on direct interaction with the indigenous people through group discussions, corner meetings and semi-structured interviews following the method by Martin [23]. A total of 14 sites were selected for the study ie Karama, Malak Mella, Landay Karama, Sam, Kaniguram, Ashpashteen, Ladha, Mordar Alagad, Salwashtai, Zawar Klai, Shak Toi, Meeshta, Kacha Langer Khel, Speena Mella (Fig. 1). Overall 197 local informants were selected as information provider belonging to different age groups in which 128 were male and 45 were female while 24 were local healers (Hakims) (Table 1). The interviewed persons were reported to be experts in the field of traditional medicines with high status in the indigenous community but most of the data was recorded from the local healers. The informants were belonging to different professions like farmers, rural herbalist (Hakeems) and housewives. To ensure the strong validity of traditional knowledge, continuous relationships were maintained with the local peoples throughout the duration of the whole survey.

**Fig. 2:**
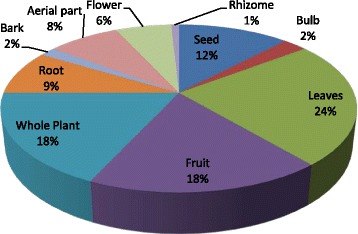
Different parts used in the study area

### Preservation and taxonomical verifications

Medicinal plants mentioned in the current survey were collected and identified by a taxonomist at the Department of Botany, Kohat University of Science and Technology. The plants were pressed for dryness, poisoned (1% HgCl_2_ solution) and were mounted on herbarium sheets. Voucher number were assigned, submitting them to the department of Botany at Kohat University, for future references by matching them for verification with the flora of Pakistan [24, 25].

### Data organization and analysis

Data compilation was carried out in MS Excel. Frequency of citation mentioned by the informants was counted for each specie. Phillips and Gentry [26] introduced the idea of relative importance of a plant through calculating the index of use value. Following is the formula for calculating use value. Use value for specie x;

$$ U{V}_x = \varSigma {U}_x/{N}_x $$

Where U_x_ is the number of use reports described by each informer for specie x while N is the total number of informers describe the specific specie x.

## Results and discussion

### Traditional knowledge and informant demographic status

The utilization of medicinal plants to combat with various ailments is as old as human civilization. It has been reported that about 20% of the whole plants found in this world are used for medicinal purposes to treat ailments in living beings [16]. In the study area, several herbal traditional recipes have been used by indigenous communities especially by the local healers, having significant role in the local health care system. They use the medicinal flora as the first aid in curing any ailment except in severe emergencies just like other parts of the country [27]. Present exploration has reported valuable traditional information about the medicinal plants from an area which is very little explored in the tribal belt of Khyber Pakhtoonkhwa [28]. Current study observed a significant decrease in the skill, faith and knowledge about traditional herbal medications due the recent introduction of allopathic medicines. Similar results were found by Sher et al. [29] in a study conducted in district Chitral. Our findings can also be justified by the other similar studies conducted earlier which have clearly shown the erosion of the precious knowledge due to modernization [1, 28, 30, 31]. Furthermore in the study region, the important medicinal flora faces some threats such as heavy grazing, uncontrollable cutting, deforestation and the collection these plants for fodder purposes by the local collectors. Similar findings were also reported by [32]. It has been reported that due to the change in socioeconomic pressure, the traditional knowledge about the folk herbal medications is slowly and gradually going towards extinction [33, 34].

Table 1 shows the demographic information of respondents while Table 2 provides the local names, part used, medicinal description and the use value of the reported plants species. A total of 197 respondents were interviewed. Medicinal knowledge was obtained from 113 while the rest of the informants were interviewed to locate the experts having the traditional knowledge in every village before conducting the ethno-medicinal survey. Informants were grouped into various demographic categories. Male informants were mostly interviewed because in the area female informant is not allowed to conduct an interview with male informant. The concept of gender segregation and veiling (Parda) is predominant in the area and according to them this is based on their religious tradition as also reported by Ahmad et al. [35]. Most of the data was gathered from the local healers (Hakims) and elder members of the community who possessed comparatively more accurate and sound traditional knowledge about the parts and recipes which improve the effectiveness of medicinal plants. That is why that the traditional herbal recipes prepared by the elder community members (traditional practitioners and collectors) are more effective then prepared by the younger ones [36, 37]. This may be attributed to the recent trend towards modernization, affecting the level and accuracy of information which transfer from generation to generation [38]. If the trend is continued for some time then it will result in the gradual disappearance of the traditional folk knowledge and will delink the current relationship between plants and human. [28]. The decreasing rate of transfer of indigenous knowledge might be due to the fact the younger generation is not taking an interest in the learning and practicing the knowledge because the indigenous societies are exposing to modernization more and more day by day [28]. While conducting the survey it was also observed that the illiterate members of the communities were more aware of the traditional knowledge as compared to educated ones. Comparatively, highly educated persons were found to be less conversant about the traditional knowledge and medicinal plants uses. Same findings were also observed in the different studies conducted in Pakistan [39] Thailand [40] and Ethiopia [41, 42]. Findings of the current study suggest that the knowledge of traditional medication is in scattered form which is to be compiled and arranged in a systematic way so as to release the knowledge from the custody of local healers and older people and to share with the other communities through published literature. No doubt those local healers and the older people in the remote areas have sufficient knowledge about the uses of medicinal plants but still they are unaware of the importance of such traditional knowledge. Special initiatives and awareness programs and projects should be designed to make fruitful strategies so as give awareness to the local communities about the importance of medicinal flora and also the importance of medicinal flora.

**Table 2:** Medicinal plant used at sub-division Ladha of South Waziristan agency. FC = Frequency of citation, UV = Use value

Family	Plant species/Family name/Voucher No	Local names (Pashto language)	Part(s) used	Medicinal uses	UV	FC
Aizoaceae	*Portulaca oleracea* L./KUSBT-511	Sormai	Aerial parts	Juice of the plant has diuretic effect and is considered valuable in treating urinary tract infections and gastrointestinal disorders such as diarrhea and dysentery. The seeds of the herb are vormifuge and demulcent.	0.87	24
Alliaceae	*Allium cepa* L./KUSBT-512	Pyoz	Bulb	Juice of the herb is used for treatment of gastrointestinal problems. It is also used as diuretic and expectorant. For the treatment of cough, bulb is wrap in cloth and worn like a necklace.	39.00	9
Amaranthaceae	*Amaranthus viridis* L./KUSBT-513	Ranzaka	Leaves	It is used as vegetable and fodder for cattle. The powder of Leaves and seeds are mixed with half weight of sugar and then a spoon of this mixture is given with black tea to the children three times daily for the treatment of constipation. Leaves are given as emollient and are used for scorpion sting, snakebite and as anthelmintic.	0.33	11
Anacardiaceae	*Pistacia chinensis* Bunge//KUSBT-514	Shrewaniay	Fruit	Galls used in native medicine, fruit oily and edible. Leaves powder is applied topically on ulcers and chronic sores. Poultice of slightly roasted Leaves is applied to inflamed swellings and rheumatic joints.	0.41	15
Arecaceae	*Nannorrhops ritchieana* (Griff.) Aitch./KUSBT-515	Mazarai	Leaves	The decoction of leaves is used as stomachic.	0.29	10
Asclepiadaceae	*Caralluma tuberculata* N.E.Br./KUSBT-516	Pamanai	Whole plant	The plant is effective in the treatment of different kinds of diseases such as dysentery, constipation, hepatitis B and C and stomach pain. It is also effective in diabetes and also used to control blood pressure.	0.67	18
Asteraceae	*Cichorium intybus* L./KUSBT-517	Speen gul	Whole plant	Plant’s decoction is effective in the treatment of liver disorders and urinary tract infections. The herb is used as tonic, laxative and diuretic. Diarrhea is also treated with the plant.	0.44	21
Asteraceae	*Lactuca serriola* L./KUSBT-518	Salad	Whole plant	Cough, bronchitis, asthma and pertussis are treated by using the herb. It is also diuretic, sedative, diaphoretic and antispasmodic.	0.87	24
Asteraceae	*Taraxacum officinale* (L.) Weber ex F.H.Wigg./KUSBT-519	Zeer gul	Whole plant	This herb is used as tonic having nutritive properties. It is strong stomachic and diuretic. The infusion of the herb is use for liver disorders. It is also efficient in dropsy, arthritis and rheumatism. Roots have laxative properties.	0.46	9
Asteraceae	*Xanthium spinosum* L./KUSBT-520	Spin aghzai	Whole plant	This herb is used medicinally as diuretic, sedative and diaphoretic.	0.27	12
Asteraceae	*Xanthium strumarium* L./KUSBT-521	Tatasai	Fruit, Roots, seeds	Powder of seeds fruits and roots are demulcent and are used in stomach disorders.	0.22	9
Berberidaceae	*Berberis lycium* Royle/KUSBT-522	De wrogha betai	Bark, Leaves, root	The roots of the plant are effective in the treatment of Skin diseases, piles and chronic diarrhea. Bark and Leaves are used as antiseptic and as tonic. To treat jaundice, mostly Leaves are used. Fruit is taken orally for the treatment of kidney problems. Decoction of root is a good remedy which is used as a purgative for the children and also used as blood purifier.	0.85	24
Brassicaceae	*Lepidium draba* L./KUSBT-523	Zangali meelay	Seed	Seeds of the plant are carminative. Mostly the plant is used as tonic and stomachic.	0.15	12
Brassicaceae	*Raphanus sativus* L./KUSBT-524	Meelay	Leaves	To cure jaundice, urinary tract infections and piles, fresh and young roots are eaten. Leaves work as diuretic and laxative.	0.67	18
Cannabinaceae	*Cannabis sativa* L./KUSBT-525	Bhangay	Flowering stems of female plants leaves,	Marijuana is the famous drug prepared traditionally from the plant. Fresh and young Leaves of the female plant are dried and grinded to make powder and is taken orally with water, milk and sweet to get cooling effect. The plant is also used to treat urinary tract diseases. The plant is also used in various medications in order to treat asthma, depression, insomnia and depression due to its sedative and analgesic effects.	0.80	10
Chenopodiaceae	*Chenopodium album* L./KUSBT-526	Spin Sormei	Whole plant	Plant is diuretic, aphrodisiac, appetizer and used as tonic. Abdominal pain is also treated with the herb. the plant is also ant helmentic and is effective in the treatment of liver disorders, jaundice. The young shoots of the plant is soaked in a glass of water for 3–5 h and then the filtrate is used orally to treat kidney pain. To remove the kidney stones, half glass of the above filtrate is mixed with equal amount to the extract of corn hair and is taken orally.	0.89	11
Compositae	*Artemisia scoparia* Waldst. & Kitam./KUSBT-527	Tarkha	Whole plant	Infusion is used as purgative. To treat burns smoke is a good remedy. Also relieve ear pain.	0.33	3
Compositae	*Aster trinervius* Roxb. ex Roxb./KUSBT-528	-	Root	Root of the plant is useful in the treatment of pulmonary infections and cough. Hemorrhage is also treated. Seed decoction is used for obstructed menstruation.	0.60	5
Compositae	*Conyza canadensis* (L.) Cronquist/KUSBT-529	-	Whole plant	Plant has diuretic and stimulant properties.	0.10	3
Compositae	*Tagetes patula* L./KUSBT-530	Zear-gullai	Fruit	Fruit is used for cooling agent, and as demulcent.	0.27	2
Convolvolaceae	*Convolvulus arvensis* L./KUSBT-531	Parwathiay	Leaves, root	The roots are dried, crushed and taken orally one or two spoon as purgative. The herb is used for skin infections. Leaves are use as poultice and as antiseptic. Leaves decoction is made up which is used two spoon daily for the regulation of abnormal menstrual cycle in woman.	0.47	4
Cucurbitaceae	*Citrullus colocynthis* (L.) Schrad./KUSBT-532	Maraghenaiy	Fruit, seeds	Juice is extracted from the plant and is mixed with sugar and is taken in dropsy. This mixture is also used externally on skin during leukoderma. Oil is extract from the seeds and used topically on skin during snake bite. Honey is mixed with the grinded fruit making Tarkha Halwa which is taken 3 to 4 teaspoon thrice a day for stomach problems and expel worms.	0.73	7
Cupressaceae	*Cupressus sempervirens* L./KUSBT-533	Servay	Fruit, root, seeds,	Fruit is given to animals to produce cooling effect. Seeds and root/decoction is used for gastrointestinal diseases.	0.27	2
Fabaceae	*Alhagi maurorum* Medik./KUSBT-534	Sobena betai	Whole plant	Plant is expectorant, laxative, anti-diarrheal and antiseptic. Exudation obtained from the branches and Leaves is used as blood purifier. Roots of the plant are dried and grinded into powder and then take two grams of the powder with water daily for 2 weeks in order to treat kidneys problems.	0.64	8
Fabaceae	*Astragalus creticus* Lam./KUSBT-535	Aghazai	Aerial parts	Arial parts of the plant have sedative effect and are used as tonic.	0.33	3
Fabaceae	*Astragalus grahamianus* Benth./KUSBT-536	Aghzai	Whole plant	The herb is a good analgesic agent and also used in the treatment of abscesses.	0.20	2
Fabaceae	*Astragalus membranaceus* (Fisch.) Bunge/KUSBT-537	Aghzia	Root	The roots of the plant are used as and vasodilator.	0.19	3
Fabaceae	*Sophora mollis* (Royle) Baker/KUSBT-538	Ghuger	Leaves, roots, seeds	Medicinally the root of the plant is used as cooling agent and as a diuretic. Leaves and seeds are used for gastrointestinal diseases urinary tract infections, eczema and are used as anthelmintic, to kill the abdominal worms.	0.69	7
Fabaceae	*Trifolium pratense* L./KUSBT-539	Jangali Shautala	Flower	Flowers heads of the herb are used to cure skin infection. The herb is a good antispasmodic and expectorant. It also has estrogenic effect helping in the control of menopausal complaints.	0.46	4
Fagaceae	*Quercus dilatata* A.Kern./KUSBT-540	Ghora tsarray	Fruit, leaves	The corns are roasted and eaten and are used as tonic.	0.26	2
Fumariaceae	*Fumaria indica* (Hausskn.) Pugsley**/**KUSBT-541	Paparie	Whole plant	Extract of the whole plant is used for the production of cooling effect. The plant is diuretic, diaphoretic.	0.68	8
Geraniaceae	*Geranium wallichianum* D.Don ex Sweet/KUSBT-542	Ranjot	Rhizome	To lower the blood pressure and to treat the leucorrhea, rhizome of the plant is used. The rhizome is also used as a source of tonic and also helps in treating rheumatism.	0.53	6
Juglandaceae	*Juglans regia* L./KUSBT-543	Matak	Fruit, root	Kernels are eaten raw to gain weight, also used as brain tonic. Root bark and Leaves are used for teeth cleaning.	0.65	5
Labiatae	*Marubium vulgare* L./KUSBT-544	Qurashka	Whole plant	The plant is used as tonic. It has also expectorant and diuretic properties and is used for pulmonary problem. Used in cold fever.	0.60	7
Labiatae	*Mentha aquatica* L./KUSBT-545	Podina	Whole plant	Decoction of the herb is used to treat digestive problems and cough. The herb is carminative and is used for flatulence.	0.80	9
Labiatae	*Mentha longifolia* (L.) L./KUSBT-546	Valanai	Leaves	Leaves decoction has carminative properties. Leaves are also employed for rheumatic pain, nausea, sickness and vomiting. Leaves of the herb are also a good remedy for the treatment of diarrhea, dysentery. Leaves powder are mixed with water and are used for stomach pain and also for cooling effect.	0.87	10
Labiatae	*Nepeta cataria* L./KUSBT-547	Chemjan betai	Whole plant	Leaves and flowering tops are dried and used as carminative agent, diaphoretic, refrigerant. Leaves are boiled and tea is prepared from it which is useful cold and fever. The tea also gives sedative effect.	0.49	7
Labiatae	*Perowskia atriplicifolia* Benth/KUSBT-548	Sansubai	Flower	Flowers are soaked in water and the water is applied to the body of the patient to produce cooling effect in fever.	0.31	3
Labiatae	*Stachy parviflora* benth./KUSBT-549	-	Leaves, stem	The bruised stem and Leaves are anthelmintic and are useful for intestinal worms	0.41	3
Labiatae	*Teucrium stocksianum* Boiss./KUSBT-550	Kastori	Whole plant	Whole of the plant water boiled and left for overnight. The water is then decanted and is taken for the treatment of cold. It is also used in cases of heart pain.	0.36	2
Labiatae	*Thymus mongolicus* (Ronniger) Ronniger**/**KUSBT-551	Marvezay	Whole plant	The herb has many medicinal properties such as it is used as carminative, as a tonic, antispasmodic. it improve poor vision. It is also used for stomach and liver problems also suppress urine and menstruation. Seeds are used as vermifuge.	0.90	11
Labiatae	*Thymus linearis* Benth./KUSBT-553	Gulapi beetai	Leaves	Leaves are used to cure cough, and asthma and expel worms from the abdomen. The Leaves have also antiseptic values.	0.31	4
Liliaceae	*Allium ascalonicum* L./KUSBT-554	Ghandana	Bulb	For the treatment of ear pain the extract of the bulb is used.	0.20	2
Liliaceae	*Allium carolinianum* DC./KUSBT-555	Jangali pyoz	Bulbs, leaves	Both of the plant parts are effective in cough and fever.	0.38	2
Liliaceae	*Tulipa lehmanniana* Merckl./KUSBT-556	Shamdai	Flower	The flower of the herbs are given to goat for increase lactation	0.27	3
Malvaceae	*Hibiscus trionum* L./KUSBT-557	Khatool	Flowers, leaves	Flowers are sunken in water and the infusion is helpful in treating skin ailments, itching. The infusion has also used as diuretic. Leaves are dried and are eaten to avoid the stomach pain.	0.47	4
Malvaceae	*Abelmoschus esculentus* (L.) Moench/KUSBT-558**.**	Bhenday	Fruit, leaves, seeds	Poultice is prepared from its Leaves and is used externally to stop irritation and treat swellings and pains. Mucilage of the fruit and seeds is a useful remedy for the treatment of irritations occurs inside the genitor urinary system.	0.40	4
Malvaceae	*Malva neglecta* Wallr./KUSBT-559	Tikalai	Seeds	Seeds are crushed and are used to cure cough and ulcer inside the bladder.	0.27	2
Malvaceae	*Malva parviflora* L./KUSBT-560	Tikalai	Leaves, root, seeds	Seeds are used in cough and in the treatment of ulcer inside the bladder. Leaf decoction is a good remedy for the expulsion of tap worm and profuse menstruation. Underground part is bruised and washed which is grinded to make fine powder then 2 g of the powder is taken and is wrapped inside the butter, eaten after dinner for sex tonic. Plant is also used as laxative.	0.53	3
Malvaceae	*Malva pusilla* Sm./KUSBT-561	Nagankai	Leaves, seeds	Leaves are applied externally to treat to scurvy and reckoned useful in piles. Seeds of the herb are used to treat skin diseases and also used in the treatment of cough, bronchitis inflammation of bladder.	0.60	6
Malvaceae	*Withania coagulans* (Stocks) Dunal/KUSBT-562	Shapianga	Fruit, leaves, seed	Dried fruit are very efficient for dyspepsia and flatulence. Fruit is crushed and the powder is taken orally daily with a glass of water to avoid the stomach ache. To treat gass trouble, 2 or 3 seeds of the herbs are taken after meal. Seeds and fruits of the plant are used to treat digestive problems, diabetes and gastritis.	0.84	11
Moraceae	*Ficus carica* L./KUSBT-563	Togha	Fruit	Fruits of the plant are eaten in constipation because of demulcent and laxative properties. Ripened fruit of the plant are crushed mixing with a glass of curd and is taken to relieve constipation. Fruit is also useful for the treatment of diabetes, urinary tract diseases and piles.	0.79	7
Moraceae	*Morus alba* L./KUSBT-564	Teeth	Fruit, leaves	Fruit of the plant have laxative properties used to relieve constipation. Leaves are expectorant and are also used to treat the fever, throat infection.	0.55	6
Moraceae	*Morus nigra* L./KUSBT-565	Thor Teeth	Fruit	Fruit has a unique test and is eaten by the local people. the fruit is useful in expelling the worm from the abdomen and useful in treating the disease of bad thorax.	0.30	3
Myrataceae	*Eucalyptus globulus* Labill./KUSBT-566	Sofaida	Leaves	the Leaves of the plant are given to diabetic patient.	0.20	2
Nitrariaceae	*Peganum harmala* L. KUSBT-567	Sponda	Aerial parts	Seeds are crushed and then they are used to treat colic, asthma, jaundice. They are also anthelmentic. Seeds are antispasmodic, narcotic. Decoction is made from the seeds so as to treat laryngitis. Fruit of the plant is useful for heart pain.	0.93	11
Oleaceae	*Olea europaea* subsp. *cuspidata* (Wall. & G.Don) Cif./KUSBT-568	Shawan	Fruit, leaves	Leaves are antiseptic. The fruit is also used as tonic.	0.47	4
Oleaceae	*Olea ferruginea* Wall. ex Aitch./KUSBT-569	Shawan	Fruit, oil	Fruit of the plant is an effective appetizer and useful treating caries of teeth and toothache. Oil of the plant is purgative. The oil is also useful in treating liver disorders and rheumatism. Powder of fruit is taken orally on empty stomach in a dose range manner of one teaspoon for forty five days. This remedy is effective in diabetes.	0.78	8
Oxalidaceae	*Oxalis debilis* var. *corymbosa* (DC.) Lourteig**/**KUSBT-570	-	Whole plant	Jaundice and dyspepsia are treating by using whole plant.	0.20	2
Oxalidaceae	*Oxalis corniculata* L./KUSBT-571	Tarvekai	Whole plant	Children eat the fresh Leaves of the herb and the local people are used the juice to treat diarrhea and stomach problems. Root decoction is used to expel worms. The extracts of the plant powder is used for scorpion sting.	0.60	5
Papilionaceae	*Medicago polymorpha* L./KUSBT-572	shapeshthlary	Leaves	Leaves have carminative properties. For the patient of blood pressure, Leaves and young and fresh shoots are very useful.	0.40	3
Papveraceae	*Papaver nudicaule* L./KUSBT-573	Jangali afeen	Fruit, leaves	Leaves and fruit of the plant have many properties like narcotic, sedative. Treating the lungs infection and bronchitis.	0.29	4
Plantaginceae	*Plantago lanceolata* L./KUSBT-574	Ispaghool	Leaves, seeds	The leaves of the plant have emollient (softening or soothing the skin) and expectorant properties. Leaves are also used as demulcent and astringent. cough and bronchitis are treated by the infusion of the Leaves. a thicken syrup is made up from the Leaves of the plant in order to alleviate the coughing in childern. seed of the plant has laxative properties. the Leaves of the plant are taken in mouth and remain there in order to avoid the toothach.	0.65	11
Poaceae	*Cymbopogon jwarancusa* (Jones) Schult./KUSBT-575	Sargarrai	Whole plant	Differnt parts of the plant are used as tonic. Decoction of the plant is useful for the patient suffering from typhoid fever.	0.27	2
Poaceae	*Cynodon dactylon* (L.) Pers./KUSBT-576	Osha	Whole plant	The grass has laxative properties and also used in asthma.	0.33	3
Pteridaceae	*Adiantum capillus-veneris* L./KUSBT-577	-	Aerial parts	Local inhabitants of the area use the fern to cure sore throat, bronchitis and cough.	0.40	3
Punicaceae	*Punica granatum* L./KUSBT-578	Nargosa	Bark, fruit	Fruit is a rich source of iron. To overcome the iron deficiency, the fruit of the plant is eaten. For nasal congestion the bark of the plant is used. Epicarp of the fruit is dried and is given to cattle for treating diarrhea.	0.91	5
Ranunculaceae	*Ranunculus muricatus* L./KUSBT-579	Chambailee	Aerial parts	Herb is diuretic. It is also used to treat urinary tract infections dysentery and jaundice. It is also useful in treating eczema, ringworm and leprosy.	0.67	6
Rosaceae	*Duchesnea indica* (Jacks.) Focke/KUSBT-580	-	Leaves	Local healers use the plant for the treatment of diarrhea and dysentery. Leaves are astringent and used as diuretic.	0.33	3
Rosaceae	*Rosa webbiana* Wall. ex Royle/KUSBT-581	Jangali Gulab	Fruit, flowers	Flower are stomachic while for the treatment of asthma the decoction of its is used.	0.21	2
Salicaceae	*Salix babylonica* L./KUSBT-582	Wala	Leaves	Leaves are crushed, and water is released from it then this water is used in 2 to 3 drops three time a day to treat ear pain.	0.13	1
Sapotaceae	*Sideroxylon mascatense* (A.DC.) T.D.Penn.**/**KUSBT-583	Gurgura	Fruit	Fruit of the plant is used to compensate the Fe deficit. The plant is attractive to honey bees. Fruit is a strong laxative having digestive properties and are also used to treat the urinary tract infections.	0.53	5
Scrophulariaceae	*Verbascum thapsus* L./KUSBT-584	Zakhta	Aerial parts	The formation and stimulation of coughing up of phlegm can be minimized by using the leaves and flowers of the plant. It is also emollient and astringent.	0.47	5
Solanaceae	*Datura metel* L./KUSBT-585	Tatsai	Flowers, leaves, roots, seeds	The plant parts are effective in fever caused due to catarrh. They are also used to remove the cerebral complications. Diarrhea and skin diseases are also treated with plant parts.	0.60	5
Solanaceae	*Datura stramonium* L./KUSBT-586	Tatisai	Fruit, leaves	Fruit and leaves are effective in Parkinson disease, bronchitis and asthma.	0.47	3
Solanaceae	*Solanum nigrum* L./KUSBT-587	Tor mrach	Fruit, leaves	Leaves are effective against sore throat, hepatitis, abdominal pain and ear pain. Berries of the plant are diuretic and are useful for piles pain. Bark of the root has laxative properties	0.67	6
Umbelliferae	*Coriandrum sativum* L./KUSBT-588	Danya	Aerial parts	Decoction of the fruit is used to relieve colic pain, bleeding piles. Fruit of the herb is stimulant, aphrodisiac, carminative and refrigerant. It also increases gastric juice secretion. Seeds of the herb are famous for increasing appetite. To treat throat infection, the decoction of the plant is used through gargling.	0.87	10
Umbelliferae	*Daucus carota* L./KUSBT-589	Gajara	Fruit, leaves, seeds	Seeds are useful in the treatment of kidney problems and uterine pain. Leave are used as vegetables Fruit and seeds are carminative, stimulant, aphrodisiac and refrigerant.	0.67	8
Urticeae	*Urtica dioica* L./KUSBT-590	Teet beetai	Aerial parts	Plant is used as tonic, diuretic, anti rheumatic, astringent.	0.53	4
Zygophyllaceae	*Fagonia cretica* L./KUSBT-591	Spelaghza	Whole plant	Plant extract is used for the patient suffering from diabetes mellitus, inflammation and scabies. The hakims of the area use the plant extract for gastrointestinal problems and pains. Fresh leaves and twigs of the plant are grinded and juice is made of them. One glass of the juice is taken daily for the treatment of gastrointestinal diseases, expulsion of abdomen worms and blood purification.	0.80	6
Zygophyllaceae	*Tribulus terrestris* L./KUSBT-592	Maklenda	Seed	Seeds of the plant are grinded and then 10 g powder is taken which is mixed with 4 g of maize flour. Then this mixture is taken 3 g after every 3 h to expel the kidney stones.	0.21	2

### Medicinal flora and its relative importance

Phillips and Gentry [26] introduced an index to calculate the relative importance of a species in term of its traditional use. This quantitative technique helps in authenticating and projecting the relative importance of species or the whole family. Different plants species showing various values in term of their use value index (Table 1). In this investigation, high use value was recorded for *Peganum harmala* (0.93) greatly contributed in treating various kinds of ailments. Other plants with high use values are *Punica granatum* (0.91) which is growing in home gardens, *Thymus mongolicus* L. (0.90), *Chenopodium album* (0.89), *Coriandrum sativum, Mentha longifolia* (0.87)*, Lactuca serriola, Portulaca oleracea* (0.87) and *Berberis lycium* (0.85), *Withania coagulans* (0.84), *Fagonia cretica* (0.80), and *Cannabis sativa* (0.80). To analyze the therapeutic potential of any medicinal plant, use value play an important contribution in determining the potent specie. Greater is the use value of any specie, greater will be its traditional importance for the indigenous community. Medicinal plant with the lowest use value was *Conyza Canadensis* (0.10), and the reason for its lower use value can be its scarcity in the area or unawareness of indigenous people about the medicinal potential of the plants specie.

### Medicinal plants uses

The present study reports 82 medicinal plants species utilized by the indigenous people in the investigated area. The reported medicinal plants obtained during the current survey were belonging to 42 families and 66 genera. Most frequently used plant’s parts were leaves followed by fruit (18%), whole plant (18%), seed (12%) and so on (Fig. 2). Many studies conducted in different ethnic communities, have reported frequently the use of leaves in traditional therapies [43–50] and the widely accepted role of leaves in traditional herbal medicines may be due to large quantity of biologically active components present inside them [51]. The consumption and harvesting of leaves and other aerial parts from medicinal plants is much better than the root for the maintenance of the specie [52]. Apart from leaves, almost all the other parts of medicinal plants such as flower, bark, stem, seed fruit are also used but the collection of that specific part depends on the requirement of the user and type of the plant species. The utilization of leaves in traditional medication may also be due to their easy availability, processing methods and minimum conservational issues [53]. Medicinal plant with multiple medicinal uses work as a strong indicator to highlight the presence of biologically active therapeutic components and other phyto-constituents and these observations and findings and such findings may prompt further research into their medicinal application [28]. Those parts of the plants which are frequently used may suggest and highlight the fact that these part may have strong medicinal values and need to further evaluate and analyze them biochemical screening and pharmaceutical evaluation so as to cross check the local and indigenous information.

**Fig. 3:**
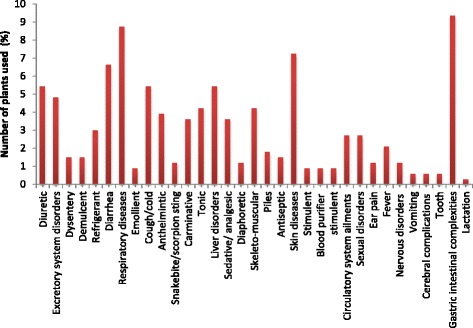
Medicinal plants used for different plants use by the indigenous people

The study indicated the use of several medicinal plants against specific diseases or category of diseases. Reported medicinal plants were used against 33 different kinds of diseases including some serious ailments like cardiac problems, hepatitis and sexual problems. Medicinal plants used for the gastrointestinal complexities and respiratory diseases showed a high incidence (9%) followed by skin diseases (7%), diarrhea (7% each), liver disorders (5%) cough and cold (5%) and so on (Fig. 3). These results indicated that the gastrointestinal problem is the common diseases occurring with high frequencies. Furthermore these gastrointestinal problems are not only common in the study area but is a common concern of the whole country [28] resulting in the higher mortality ratio if the diseases are not treated promptly and quickly [54].

Indigenous communities use to cultivate important medicinal plants in their home gardens including *Cannabis sativa, Raphanus sativus, Mentha aquatica, Allium ascalonicum* and *Peganum harmala* etc. Mostly the people collect the medicinal plants from open area because the area is so much diversified with several medicinal plants. Medicinal plants frequently used include *Peganum harmala, Punica granatum, Thymus mongolicus, Chenopodium album, Coriandrum sativum*, *Mentha longifolia, Lactuca serriola*, *Portulaca oleracea*, *Berberis lycium*, *Withania coagulans* and *Fagonia cretica* etc. current exploration has also found the over collection of two most important economical valuable species ie *Caralluma tuberculata* and *Nannorrhops ritchiana* [55] which have the capacity to cultivate [56] so as to restore their ecological role and because these two taxa are under great threat due to their over consumption. In a study conducted by Adnan et al. [28] The same species were recorded with facing the same threats.

Current investigation recorded the recipes in the form drying and macerating into powder, boiling as tea, juicing and pulsation into paste are the common administration methods observed in the study area. (Table 2) [28]. Deeba [57] reported that grinding or crushing and boiling as tea are the most common and efficient methods for the extraction of active ingredients. During the survey it was mentioned by the traditional healers that the use of complex medicines which is formed by the combination of two or more plant parts is more potent medicinally as compared to the medicines which is prepared from single plant species which is an agreement with the findings of [58]. The use and better efficacy of those recipes which are formed from more than one medicinal plant can be attributed to the synergistic or additive effects [59]. The way which is adopted for the preparation of drug differ from individual to individual in which the same plant material is prepared in different way for the same ailment. For example, the aerial parts of *Peganum harmala* are used against colic pain, jaundice, asthma, spasm and as narcotic. The decoction is made from its seeds is used for the treatment of laryngitis (Table 2). These findings are running parallel with the findings of the study conducted by Ullah et al. [31] in which the same ailments were treated by the plant but instead of aerial parts, fruit was used. Fruit of *Punica granatum* is used to overcome the iron deficiency. For nasal congestion, the bark of the plant is used. Epicarp of the fruit is dried and is given to cattle for treating diarrhea (Table 1). Kayani et al. [39] reported the uses of *Punica granatum* powdered form, prepared from fruit, bark and leaves for the treatment of whooping cough from Gallies, Abbotabbad, Pakistan. Similarly in the study the *Thymus mongolicus,* is used as carminative, tonic and antispasmodic. It improves poor vision. It is also used for stomach and liver problems also suppress urine and mansturation. Seeds of the plant are used as vermifuge. Farooq et al. [60] reported the uses of *Thymus mongolicus* as antispasmodic, carminative, tonic and is given in weak vision, complaints of the stomach and liver, suppression of urine and menstruation. *Chenopodium album* is as diuretic, aphrodisiac, appetizer and used as tonic. Abdominal pain is also treated with the herb. the plant is also anthelmintic and is effective in the treatment of liver disorders, jaundice. Decoction of young shoots is used orally to treat kidney pain. In the current study *Lactuca serriola* is used as sedative, diuretic, diaphoretic, antispasmodic and expectorant. Findings about the medicinal uses of *Lactuca serriola* reported in our study are going parallel to the finding of Ullah et al. [31]. In the same way, study conducted by Kayani et al. [39] it was found that *Lactuca serriola* whole plant is used as expectorant and also utilized for the treatment of cough, phthisis, bronchitis and asthma. The similarities observed in the cross cultural uses of the traditional herbal remedies indicate the biological potential of the documented flora. To minimize the effect of the remedy’s astringent taste, different liquids such as water, sugar, juices, oil are also mixed with the processed plants parts so as to avoid the bitter taste of the remedies. To minimize the relative potency of the recipe, different the above mentioned vehicles are used which further dilutes the drug [61].

Despite the fact that folk use the plants for several infections but except few medicinal plants species, most of the plants documented in the current investigation are not still analyzed for their detailed pharmacological potential. For instance *Chenopoduim album* has been screened out comprehensively for its phytochemical anthelminthic potential and further work is also in progress [62]. Khan et al. [63] investigated *Peganum harmala* for antimicrobial potential obtained from Margalla Hills, Islamabad. Antimicrobial activity of different plants’ extracts of *Datura stramonium* have been evaluated for their respective potential, from the region of Khyber Pakhtoonkhwa, Pakistan [64]. Similarly *Withania coagulans* has been investigated pharmacognostically in detail by researchers [65]. Esra et al. [66] analyzed *Cannabis sativa* for its pharmacological potential against fungal and bacterial diseases and was found the best against the targeted diseases. But still there are varieties of medicinal plants such as *Sophora mollis, Thymus mongolicus* and *Tulipa lehmanniana* and so many others which need detailed pharmacological and critical toxicological studies in order to make safe and effective utilization of the herbal products. The discovery of new biological active constituents should be focused during such phytopharmacological investigations.

## Conclusions

Ladha is a remote area where the local people still rely on traditional herbal therapies for their primary health care services. In the study area the traditional knowledge is in custody of elder community members and local herbalists. The study reports several important medicinal plants having significant contribution in the treatment of different diseases. Our study has a contribution in the documentation of traditional knowledge because the knowledge is losing its originality day by day due to exposure to modernization. The study highlights the need exploration of pharmacological, toxicological, phytochemical and microbiological studies of the reported medicinal plants to make the better and effective use of the plants. Present investigation highlights several threats including heavy grazing pressure, cutting activities, deforestation which affecting the sustainability and declining the population of the local flora. Apart from this the study area is suffering from terroristic activities and “War on Terror” is going on, which is also a great issue to be addressed. Research projects should be designed to analyze comprehensively the conservation status and threats to the flora in the study area. While designing research management plans and strategies, the existing ecological and other cultural matters should be documented and addressed.
